# Cerebral localization of higher functions: Memory-related anatomic structures

**DOI:** 10.1590/1980-57642020dn14-010012

**Published:** 2020

**Authors:** Eliasz Engelhardt

**Affiliations:** 1 Cognitive and Behavioral Neurology Unit, INDC – CDA-IPUB – UFRJ – Rio de Janeiro, RJ, Brazil.

**Keywords:** memory, anatomical structures, diencephalon, hippocampus, neocortex, memória, estruturas anatômicas, diencéfalo, hipocampo, neocórtex

## Abstract

The nature of memory and the search for its localization have been a subject of interest since Antiquity. After millennia of theoretical concepts, shifting from the heart to the brain, then from the ventricles to solid parts, the core memory-related structures finally began to be identified through modern scientifically-based methods at the diencephalic and cortical (hippocampal and neocortical) levels, mostly in the late Modern period, culminating in the current state of knowledge on the subject.

## RESUMO

A natureza da memória e a busca de sua localização tem sido objeto de interesse desde a Antiguidade. Após milênios de conceitos teóricos, mudando do coração para o cérebro e daí dos ventrículos para as partes sólidas, as estruturas centrais relacionadas com a memória finalmente começaram a ser identificadas através de métodos modernos com base científica, nos níveis diencefálico e cortical (hipocampal e neocortical), principalmente no período Moderno tardio, aproximando-se do estado atual do conhecimento sobre o tema.

The nature of memory (function and failure), as well as the search for its localization, have been an object of interest since Antiquity.^1^^,^ 2 The earlier Western civilizations (e.g., ancient Egyptians) had elected the heart as the central organ, while the Greeks were divided on this matter, cardiocentric vs encephalocentric. Thus, Empedocles, Democritus, Aristotle, Diocles, Praxagoras favoured the heart, whereas Alcmaeon, Pythagoras, Plato, Herophilus, Erasistratus, Rufus, and Galen chose the brain.^1^^-^ 3 Finally, the brain prevailed. The description of the ventricles in the human brain by Herophilus and Erasistratus (IV-III century BC) created a place to locate the soul and mind. Much later, Nemesius of Emesa (ca. 390 AD) located the functions of the human mind – senses, reasoning, and memory – in these different ventricles, proposing the “doctrine of ventricular localization of mental function” (possibly around the end of the IV century AD [first translation published in 1538]).^2^^-^ 4 This view, in a descriptive manner, prevailed for many centuries, being expanded by Albertus Magnus (ca. 1193-1280), whose book illustrated the cerebral cavities (ventricles) schematically, apparently for the first time (1506, chapter XIII – posthumous release).^3^^,^ 5 ^-^ 7 Nemesius, Albertus and other authors of their time who localized the mind and its faculties within these cavities placed memory in the posterior ventricle (cerebellar ventricle) (4^th^ ventricle), where it remained until the XVII century (Renaissance). At this point, the faculties were understood to be in the solid parts of the cerebrum, as proposed by Johannes Jakob Wepfer (1658), and a short time later by Thomas Willis (1664).^3^ During the entire Middle Ages (V-XIV century) the ventricles remained the center of the theories relating mind and brain, with the latter housing the faculty of memory.^2^^,^ 6

The long preceding period of these initial localization theories gave way to a phase of modern, more concrete and scientifically-based facts, in a bid to relate memory failure (amnesia) to solid brain structures, which took place mainly in the XIX century, extending into the XX century (late Modern period). At this time. numerous anatomical brain structures were found to be related to this dysfunction (and function), furthering understanding on the matter and culminating in the present state of knowledge.

## MEMORY-RELATED ANATOMICAL BRAIN STRUCTURES

The memory-related brain structures known today can be divided into diencephalic and cortical (hippocampal and neocortical), and involve the core components of the declarative memory circuits. The nuclei with modulatory functions, such as the cholinergic Meynert’s basal nucleus, will not be addressed. The development of this knowledge is outlined in the Table. It is meaningful to stress that most of the considered structures have been already described a long time before determining their memory function, a point that will not be focused in the present paper.

**Table:** Anatomical structures first related to memory (current names in square brackets).

structure	• 1^st^ related to memory
thalamus (nuclei)	• anterior tubercle [anterior nuclei] (Gudden,1896)^17^ • nucleus parafascicularis [parafascicular nucleus], submedial nucleus [submedius nucleus], nucleus reuniens [reuniens nucleus], medial thalamic nucleus (part) [medial mediodorsal nucleus] (Gamper, 1928)^19^
mammillary bodies	• mammillary bodies (Gudden, 1896)^17^
mammillothalamic tract	• bundle of Vicq d'Azyr [mammillothalamic tract] (Gudden, 1896) 17
hippocampus	• hippocampal region (stroke) (Bechterew, 1900)^20^ • hippocampal region (surgery) (Scoville and Milner, 1957)^22^ • hippocampus proper (Zola-Morgan et al., 1986)^24^
dentate gyrus	• dentate gyrus (included in hippocampal region) (Scoville and Milner, 1957)^22^ • dentate gyrus (Baker et al., 2016)^25^
fornix	• fornix (Gudden, 1896)^17^
cerebral neocortex	• outer surface of brain [cerebral cortex] (speculative) (Willis, 1664)^28^ • associative cerebral neocortex (Penfield, 1968)^33^

## DIENCEPHALIC MEMORY-RELATED STRUCTURES

The modern studies of memory were triggered by Carl Wernicke (1848-1904) and Sergei Sergeievich Korsakow (1854-1900). First, Wernicke described three acute cases (two with alcoholic cause), without memory impairment, which evolved to death. The autopsy revealed punctiform hemorrhages in the floor of the 3^rd^ and 4^th^ ventricles, and in the acqueductal region. For this condition, he proposed the diagnosis of ‘Superior Hemorrhagic Poliencefalitis’ (1881).^8^ Later he described chronic alcoholics with severe loss of retention memory and retroactive amnesia, spared memory storage [remote], besides confabulation and temporal disorientation. No autopsy was reported (1900).^9^ Contemporaneously, Korsakow described a disorder related to alcoholism (1887-1891), with symptoms comprising disorientation, amnesia characterized by quickly forgotten recent facts [anterograde amnesia], preserved remote events [long-term memory], loss of memory from onset to a period preceding the disease [retrograde amnesia], and pseudo-reminiscences [confabulation].^10^^,^ 11 The condition was named after him as ‘Korsakow’s syndrome’ or ‘Symptom complex of Korsakow’ by Friedrich Jolly (1887).^12^ He published one case of this type with autopsy verification, where no macroscopic or microscopic abnormalities of the brain were observed.^13^

Their studies were not able to objectively identify the underlying anatomic structures. Wernicke’s pathological findings would only be later understood, retrospectively.^8^ Korsakow believed the memory failure might be explained by the disorganization of association fibers connecting the nervous cells of the cerebral cortex,^14^ but without supporting evidence.

The close relationship between both syndromes was not recognized by their authors. However, Adolf Elzholz and Karl Ludwig Bonhoeffer identified this relationship, recognizing the strong relation between the two syndromes, regarding them as part of the same pathological process.^12^^,^ 15 ^,^ 16

Two publications that followed Korsakow’s are worthy of note, as they furthered understanding on the subject, namely, the studies of Hans Gudden (1866-1940) and of Eduard Gamper (1887-1938), which provided entirely novel anatomical information. Gudden presented clinical and pathological data on five patients with severe chronic alcoholism who died in the acute or chronic phase of the condition (1896). They presented a clinical picture resembling that of Korsakow’s and pathological examination showed lesions compatible with Hemorrhagic encephalitis, affecting the walls of the 3^rd^ ventricle, superior brainstem, and 4^th^ ventricle, and also lesion of the anterior tubercle of the thalamus, and marked atrophy of the mammillary bodies, while the fornix and Vicq d’Azyr bundle showing abnormal changes in one of the cases.^17^^,^ 18

At a later date (1928), Gamper held a conference about chronic alcoholics who died in the acute, subacute or chronic phase with the clinical manifestation of Korsakow’s psychosis, with or without Polioencephalitis haemorrhagica (Wernicke’s). Neuropathological examination showed lesions of the brainstem, mammillary bodies, and thalamic nuclei (parafascicularis, submedial, reuniens, and medial part of the medial thalamic nucleus). He held that the mammillary bodies constituted an important nodal point of the vegetative mechanism underlying the psychic processes of the amnesic syndrome, considering their strong connections, on one side with the midbrain and the thalamus (and through this certainly to the cingulate gyrus) and on the other side with the Ammon’s formation by way of the fornix.^18^^,^ 19

Apparently Gudden was the first author to relate the amnesic symptoms of his cases with anatomic structures, such as the mammillary bodies and related tracts (fornix and Vicq d’Azyr bundle), and the anterior tubercle of the thalamus. Gamper endorsed and extended Gudden’s finding, and proposed a circuit formed by the connections of the mammillary bodies, thalamus, and cortical structures (hippocampus and cingulate gyrus), related to memory failure.

The findings of Gudden and those of Gamper, with the suggestion of the above-mentioned circuit, can be viewed as possible precursors of the circuit described later by James Papez (1937), initially proposed as a mechanism of emotion.

The cited studies, particularly those of Gudden and Gamper, have revealed the critical role of diencephalic structures in memory function, prompting further search for distinct brain structures and neural circuits underpinning the memory process.

## HIPPOCAMPAL MEMORY-RELATED STRUCTURES

Vladimir Michailovich Bechterew (1857-1927) presented a case with severe memory weakness [amnesia], falsification of memories (pseudo-memories) [confabulation], and apathy (1900). The autopsy revealed softening of the cerebral cortex in the region of the anterior (gyrus uncinatus) [uncus], and the internal part of both temporal lobes (gyrus cornu Ammonis) [hippocampus proper – fascia dentata – subiculum (according to Bechterew, 1899)] and adjacent parts. He commented that the case presented the characteristic symptom complex of Polyneuritic psychosis [Korsakow’s] that could also occasionally manifest in organic lesions of the cerebral cortex.^20^^,^ 21

Bechterew’s report was the first relating memory impairment with lesion of the hippocampal region.

About half a century later, this feature was confirmed by William Beecher Scoville (1906-1984). He performed bilateral medial temporal resection for refractory epileptic seizures and other conditions. One of these patients, later known as the HM case, had surgical removal of the medial surface of the temporal lobes, anterior ⅔ of the hippocampus and the [para]hippocampal gyrus bilaterally, as well as the uncus and the amygdala. The patient was assessed by Brenda Milner who detected severe loss of anterograde memory and partial loss of retrograde memory, whereas early memories appeared normal and technical skills also remained intact. These findings indicated the importance of the hippocampal region [hippocampus and related structures] for the normal functioning of anterograde memory.^22^ It is important to stress that the removed hippocampal region included the hippocampus proper, dentate gyrus and subiculum, as well as adjacent temporal lobe structures, as revealed later by neuroimaging and autopsy studies.^23^ Further studies proceeded in the ensuing decades to distinguish the role of the various components of the hippocampal region in memory processing – the hippocampus proper,^24^ dentate gyrus^25^ and subiculum complex, and the immediately related regions, namely the perirhinal, entorhinal and para-hippocampal cortical areas.^26^

## NEOCORTICAL MEMORY-RELATED STRUCTURES

The first to suggest the involvement of the cerebral cortex with memory handling was Willis (1664), albeit in a conjectural manner.^27^^,^ 28 Further speculative studies followed. New approaches established that the cerebral neocortex represented the seat of storage of long-term memory. This was examined experimentally by Karl Lashley, who inferred that widely dispersed neuronal assemblies represented memories or ‘engrams’ (1950) and also proposed theoretically by Friedrich Hayek, who formalized it in large-scale cortical networks (or “maps”) representing all experience acquired through the senses (1952).^29^

The HM case, as seen earlier, in which long term-memory (‘early memories’) was preserved after the surgery,^22^ supported the belief that its area of storage lay outside the regions removed (i.e., extra-hippocampal), presumably located in the spared association neocortex.^30^^,^ 31

The first to demonstrate experimentally that the human cerebral cortex was related to memory was Wilder Graves Penfield (1891-1976). He stimulated the cortex of awake patients who underwent surgery for epilepsy treatment and stated (1959): “There is an area of the surface of the human brain where local electrical stimulation can call back a sequence of past experiences”.^32^ The stimulated regions mentioned were the superior temporal gyrus and the temporo-occipital area, mainly on the left side, where auditory and visual past experiences [identified as stored long-term memory] were elicited (1963).^33^^,^ 34 These areas are now acknowledged as associative neocortex.

Today, it is known that the frontal and temporo-parieto-occipital associative neocortical areas constitute the sites where long-term memories are presumed to be stored.^31^^,^ 35

## CONCLUSION

The nature of memory function, as well as the search for its localization, has been a subject of interest since Ancient times. After millennia of theoretical concepts, the core memory-related structures began to be identified, mostly in the late Modern period (XIX and XX centuries), through modern scientifically-based methods. First the diencephalic, then the hippocampal structures were found, and finally the neocortical structures, culminating in the current state of knowledge.
